# Serologic Vaccination Response after Solid Organ Transplantation: A Systematic Review

**DOI:** 10.1371/journal.pone.0056974

**Published:** 2013-02-22

**Authors:** Isabella Eckerle, Kerstin Daniela Rosenberger, Marcel Zwahlen, Thomas Junghanss

**Affiliations:** 1 Section of Clinical Tropical Medicine, Department of Infectious Diseases, University Hospital Heidelberg, Heidelberg, Germany; 2 Institute of Virology, University of Bonn Medical Centre, Bonn, Germany; 3 Institute of Social and Preventive Medicine, University Bern, Bern, Switzerland; Children's Hospital Boston/Harvard Medical School, United States of America

## Abstract

**Background:**

Infectious diseases after solid organ transplantation (SOT) are one of the major complications in transplantation medicine. Vaccination-based prevention is desirable, but data on the response to active vaccination after SOT are conflicting.

**Methods:**

In this systematic review, we identify the serologic response rate of SOT recipients to post-transplantation vaccination against tetanus, diphtheria, polio, hepatitis A and B, influenza, *Streptococcus pneumoniae*, *Haemophilus influenzae*, *Neisseria meningitides*, tick-borne encephalitis, rabies, varicella, mumps, measles, and rubella.

**Results:**

Of the 2478 papers initially identified, 72 were included in the final review. The most important findings are that (1) most clinical trials conducted and published over more than 30 years have all been small and highly heterogeneous regarding trial design, patient cohorts selected, patient inclusion criteria, dosing and vaccination schemes, follow up periods and outcomes assessed, (2) the individual vaccines investigated have been studied predominately only in one group of SOT recipients, i.e. tetanus, diphtheria and polio in RTX recipients, hepatitis A exclusively in adult LTX recipients and mumps, measles and rubella in paediatric LTX recipients, (3) SOT recipients mount an immune response which is for most vaccines lower than in healthy controls. The degree to which this response is impaired varies with the type of vaccine, age and organ transplanted and (4) for some vaccines antibodies decline rapidly.

**Conclusion:**

Vaccine-based prevention of infectious diseases is far from satisfactory in SOT recipients. Despite the large number of vaccination studies preformed over the past decades, knowledge on vaccination response is still limited. Even though the protection, which can be achieved in SOT recipients through vaccination, appears encouraging on the basis of available data, current vaccination guidelines and recommendations for post-SOT recipients remain poorly supported by evidence. There is an urgent need to conduct appropriately powered vaccination trials in well-defined SOT recipient cohorts.

## Introduction

The numbers of solid organ transplant (SOT) recipients have substantially increased in recent decades. Transplant rejection rates decreased with improved immunosuppression and quality of life increased with better post-transplant care. Donor- and recipient- derived, opportunistic, nosocomial or community acquired infections including newly emerging infectious diseases and malignancies remain a problem, however [1], [2]. Most infections in SOT recipients are associated with high morbidity and mortality compared to immunocompetent individuals. Furthermore, more powerful immunosuppressive drugs, such as biologic agents are changing the pattern of infection with new, previously unknown infection risks [3], [4].

Vaccination is the most efficient and cost effective intervention to prevent infectious diseases in healthy persons [5]. The response to vaccines with subsequent protection against infectious diseases, however, is depending on a functioning immune system.

Despite current recommendations emphasizing the importance of vaccinating SOT candidates prior to transplantation, reality tells a different story: vaccine coverage studies have demonstrated that this goal is far from being achieved. In the pre-transplantation phase practical problems of timely vaccination are difficult to overcome and concerns by both patients and doctors about vaccine-induced side effects are the main reason for the reluctant vaccination uptake in the post-transplantation period [6], [7].

Guidelines and recommendations for vaccination of SOT recipients are poorly supported by evidence and are largely extrapolated from what is known for healthy persons. For new immunosuppressive regimens and recently approved vaccines, recommendations are solely based on theoretical considerations.

We systematically review published data on the vaccination response of SOT recipients to summarize current knowledge and to identify areas where research is needed.

## Methods

### Search Strategy

We systematically reviewed published work in accordance with the PRISMA guidelines [8]. We performed an electronic search with the following medical subject heading (MESH) terms: “immunization”, “vaccination” and “organ transplantation” in PubMed, Embase and Cochrane Central Register of Controlled Trials without time restriction till end of December 2011. The search was restricted to articles on human research and English language. References of the papers identified and of relevant reviews and books were additionally searched.

### Trial Selection

We included original research papers published in peer-reviewed scientific journals reporting results on adult or paediatric SOT recipients that were vaccinated with currently licensed vaccines (see Table 1). We included all studies in which vaccination appeared warranted as defined by those who designed and conducted the study. This definition included studies where SOT recipients were considered unvaccinated in the pre-transplant era, SOT recipients whose vaccination status was not updated before transplantation, SOT recipients with negative antibody status or with unknown vaccination status.

**Table 1:** Criteria and definitions for study selection and data analysis.

**A**	
Inclusion criteria	Active vaccination
	Licensed vaccine still in use
	Adult or paediatric SOT recipients on immunosuppressive medication
Exclusion criteria	Vaccination of solid organ transplant candidates or donors
	Experimental vaccines
	Experimental adjuvants
	Comparative trials on routes of vaccine application
	Vaccines out of use
	Pandemic vaccines
	Humoral (serologic) response not assessed
	Key data missing (number of vaccines, rate of response, and time of vaccination)
	No primary data given (reviews, editorials, guidelines, letter to the editor and comments on other articles)
	Case reports
	Conference reports
	Trials on prevention of HBV recurrence in liver graft recipients with a history of HBV infection or anti-HBc positive grafts.
**B**	
***Term***	***Definition***
SOT recipients	Recipient of an organ graft receiving immunosuppression
Routine vaccination	Vaccines which are licensed and currently in use
Indication for vaccination	Vaccination appeared warranted as defined by those who designed and conducted the trials (SOT recipients considered unvaccinated in the pre-transplant era, vaccination status was not updated before transplantation, negative antibody status or unknown vaccination status)
Positive response	Humoral (serological) vaccine response as rate of seroconversion, if available, or as serological response defined as “protective” by the authors
Short-term response*	Positive response >2 weeks and ≤3 months post-vaccination
Long-term response*	Positive response ≥12 months post-vaccination

Inclusion and exclusion criteria (A) and definitions used for study selection and data analysis (B).

* If a shorter time interval was accepted exceptionally, this is indicated in the text or by a footnote.

Serologic immune response to vaccination had to be assessed at least once after vaccination. All types of vaccines that are currently in use independent of the vaccination schedule, combination and dosing were accepted. No restriction regarding study design was applied.

We excluded studies on vaccination of transplant candidates or organ donors, experimental vaccines or adjuvants, comparative trials on routes of application, trials on vaccines that are out of use and trials on pandemic vaccines. We excluded articles where humoral (serologic) response) was not assessed, key data were missing or not extractable (number of SOT recipients vaccinated, rate of response, and time of vaccination), when no primary data were given (all reviews, editorials, guidelines, letter to the editor and comments), case and conference reports. Further, we excluded trials on vaccination for prevention of hepatitis B recurrence due to HBV-related liver disease, transplant recipients with a history of HBV infection or anti-HBc positive grafts. If the same group of SOT recipients was described in more than one article, only the report with the more detailed data presentation was included.

**Figure 1:**
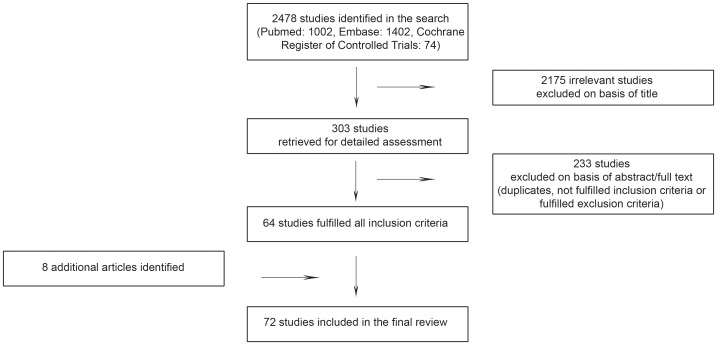
Flowchart of study selection.

All articles obtained in the search were reviewed on the basis of the title in a first step, if the title was unclear, the abstract was additionally reviewed (exclusion step one). After subtraction of duplicates, articles were checked for inclusion criteria on the basis of the abstract or the full text (exclusion step two). Articles not fulfilling the inclusion criteria were excluded (exclusion step three). Hard copies of the articles fulfilling all inclusion criteria were obtained and the data extracted with a data extraction sheet. Additionally, the reference list of relevant articles and reviews were searched (for data flow, see Figure 1). Both authors performed all selection steps independently.

### Data Extraction

From all articles remaining after exclusion step two the following data were extracted: Type of trial, type of SOT, age-group of SOT recipients (adult/paediatric), type of vaccine, vaccination schedule (number of doses and timing), number of SOT recipients and controls vaccinated, proportion of subjects (SOT recipients and, if given, controls) with vaccine response, time of follow up (defined time interval between vaccination and serological tests), cut-offs used for definition of vaccine response and immunosuppressive regimen.

### Data Analysis

As outcome of interest we choose the serologic response to vaccination and report the overall proportion of SOT recipients with vaccine response at two time points after vaccination. For our analysis the vaccination response defined by the authors of the individual studies was accepted as valid. Short-term response was defined as the humoral (serological) vaccine response measured as rate of seroconversion where this information is available, or as serological response defined as “protective” by the authors two weeks to three months after vaccination. Long-term response was defined as humoral (serological) vaccine response measured after 12 months or longer. If a shorter time interval was exceptionally accepted as long-term response, this is indicated (Table 1).

For detailed analysis, we grouped all articles obtained in the following categories: category A includes all studies on vaccination against tetanus, diphtheria, and polio. Category B includes vaccination against viral hepatitis A and B (HAV and HBV). Category C includes vaccination against seasonal influenza; category D includes vaccination against bacterial pathogens (*Streptococcus pneumonia*, *Haemophilus influenzae,* and *Neisseria meningitides*). Category E includes all live virus vaccines (mumps, measles, rubella and varicella) and category F includes vaccination against pathogens indicated in certain geographical areas (tick-borne encephalitis virus and rabies virus) (Table 2).

**Table 2:** Categories of vaccinations used and rationale for grouping.

Category	Vaccinations against	Rationale for grouping
A	Tetanus, Diphtheria, Polio	Inactivated vaccines which are frequently given as combined vaccination, part of regular childhood immunization scheme
B	Hepatitis A, Hepatitis B	Inactivated vaccines against viral hepatitis, available as combination vaccine
C	Influenza	The only vaccine which is indicated yearly and has a seasonally changing antigen composition, inactivated trivalent vaccine includes three different antigens (H1N1, H3N2 and B)
D	*Streptococcus pneumoniae, Haemophilus influenzae, Neisseria meningitides*	Inactivated vaccines against bacterial pathogens*Streptococcus pneumoniae*: available either as polysaccharide or conjugate vaccine, response includes a large number of antigens (7 or 23 serotypes) which differ in their immunogenicity
E	Varicella, Mumps, Measles, Rubella	Attenuated live virus vaccines which are given in childhood, available as combination vaccine
F	Tick-borne encephalitis, Rabies	Vaccines which are indicated for certain geographical areas, not part of the basic vaccination scheme

### Forest Plot

We calculated the percentage of seropositive individuals at follow-up by dividing the number of patients with positive response by the total number of patients with exact binomial 95% confidence intervals (CIs) separately for each vaccination group in each study and displayed these in forest plots. Studies which only reported calculated percentage numbers were not included. For influenza forest plots, only studies which assessed response to every one of the three single antigens (H1N1, H3N2 and B) were included in the forest plot. We used meta-regression to compare response proportions, for example between adult and paediatric transplant recipients. The standard error for the response proportion in a study was derived from the lower and upper confidence limit of the response proportion. Serological responses in transplant patients were compared with those of comparison groups when available in each study with the use of the risk difference (RDs) and 95% CIs either assuming a common-effect by using the Mantel-Haenszel method or by using the DerSimonian and Laird random-effects meta-analysis as implemented in the “metan” command in Stata [9]. We quantified between-trial heterogeneity with use of the I-squared statistic [10].

## Results

In total, we obtained 2478 articles in the search (Figure 1). Based on title, 303 potentially suitable articles of those were selected. Remained articles which were checked for duplicates, then for inclusion/exclusion criteria on the basis of abstract, or, if not all the necessary information was provided in the abstract, on the basis of the full text. From these articles, in total 233 articles were excluded.

64 articles obtained through the initial search fulfilled all inclusion criteria. By searching the reference lists of relevant articles, reviews and books, 8 papers were additionally identified. In total 72 papers were included into the review.

Of the trials that fulfilled the inclusion criteria (Table 3), publication date ranged from 1980 to 2011. There were prospective controlled (n = 32), prospective uncontrolled (n = 30), retrospective (n = 4), and randomized (n = 5) trials and, although initially defined as exclusion criteria but due to its relevance, one case report (n = 1) included in the review. Some trials used more than one trial design within the same publication on different patient cohorts. Of the five randomized trials, three randomized to the type of vaccine used (two for pneumococcal conjugate vaccine vs. polysaccharide vaccine [11], [12] and one for influenza vaccine subunit vs. virosomal vaccine [13]), one trial on influenza vaccination was randomized for the immunosuppressive regimen (calcineurin-inhibitor vs. sirolimus [14]) and one trial which primarily observed graft rejection was randomized to intervention (single dose influenza vaccination vs. no vaccination [15]). In total, 65 trials were investigating inactivated vaccines in both adults and children and seven trials investigated live attenuated vaccines solely in paediatric SOT recipients. Inclusion of a control group was highly variable with control groups entirely missing for HBV and for live vaccines. At the opposite end the majority of influenza vaccine trials had control groups.

**Table 3:** Characteristics and findings of all studies included (n = 72).

Category A: Tetanus							
First author	Year	Design	Patients	Short-term response SOT (%)	Short-term response HC (%)	Long-term response SOT (%)	Vaccine, Vaccination schedule
Girndt	1995	pc	RTX, A	6/7 (85%)	13/13 (100%)	–	T, triple dose (month 0, 1, 6)
Enke a)	1997	pu	RTX, P	42/42 (100%)	–	42/42 (100%)	Td, single dose
Huzly a)	1997	pc	RTX, A	150/150 (100%)	95/96 (99%)	–	Td, single dose
Ghio	1997	pu	RTX, P	33/33 (100%)	–	–	TD, single dose
Pedrazzi a)	1999	pu	RTX, P	35/35 (100%)	–	34/35 (97.1%)	Td, single dose, booster for non-responders
Puissant-Lubrano a)^1^	2010	pu	RTX, A	4/13 (30.8%)	–	–	T, single dose combined with influenza vaccine
Puissant-Lubrano b)^2^	2010	pu	RTX, A	16/26 (61.5%)	–	–	T, single dose combined with influenza vaccine

Category A: Diphtheria							
First author	Year	Design	Patients	Short-term response SOT (%)	Short-term response HC (%)	Long-term response SOT (%)	Vaccine, Vaccination scheme
Enke b)	1997	pu	RTX, P	N/N (95%)	–	N/N (76%)	Td, single dose
Huzly b)	1997	pc	RTX, A	145/164 (88.5%)	102/106 (96.2%)	34/55 (62%)	Td, single dose
Pedrazzi b)	1999	pu	RTX, P	33/35 (94.3%)	–	31/35 (88.6%)	Td, single dose, booster for non-responders
Ghio	1997	pu	RTX, P	31/33 (94%)	–	–	TD, single dose

Category A: Polio							
First author	Year	Design	Patients	Short-term response SOT (%)	Short-term response HC (%)	Long-term response SOT (%)	Vaccine, Vaccination scheme
Huzly c)	1997	pc	RTX, A	150/164 (91.6%)	47/48 (97.9%)	NA/55 (% NA)	IPV, single dose

Category B: Hepatitis A							
First author	Year	Design	Patients	Short-term response SOT (%)	Short-term response HC (%)	Long-term response SOT (%)	Vaccine, Vaccination scheme
Dumot	1999	pu	LTX, A	0/8 (0%)	–	–	HAV, double dose (0, 2 months)
Günther a)	2001	pc	LTX, A	37/38 (97.4%)	27/27 (100%)	16/27 (59.3%)	HAV, double dose (0, 6 months)
Günther b)	2001	pc	RTX, A	28/39 (71.8%)	27/27 (100%)	6/23 (26.1%)	HAV, double dose (0, 6 months)
Arslan	2001	pu	LTX, A	6/23 (26%)	–	–	HAV, double dose (0, 6 months)

Category B: Hepatitis B							
First author	Year	Design	Patients	Short-term response SOT (%)	Short-term response HC (%)	Long-term response SOT (%)	Vaccine, Vaccination scheme
Wagner	1992	pu	HTX, A	6/86 (6.9%)	–	–	rHBV, triple dose (0, 1, 6 months)
Lefebure	1993	pu	RTX, A	14/39 (36%)	–	–	rHBV, quadruple dose (0, 1, 2, 6 months)
Berner	1993	pu	LTX, A	6/21 (29%)	–	–	rHBV, quadruple dose (0, 1, 2, 12 months)
Chalasani	1998	r	LTX, A	3/45 (6.7%)	–	–	rHBV, triple dose (0, 1, 3 months)
Duca	2001	pu	LTX, P	40/47 (85%)	–	–	rHBV, triple dose (0, 1, 6 months), additional booster for non-responders
Lin	2006	pu	LTX, P	24/30 (80%)	–	–	Primary vaccination in infancy, followed by booster vaccinations post-transplant
Ni a)	2008	pu	LTX, P	7/11 (63.6%)	–	–	Primary vaccination in infancy, single booster
Ni b)	2008	pu	LTX, P	4/4 (100%)	–	–	Primary vaccination in infancy, single booster followed by additional booster to non-responders

Category C: Influenza						
First author	Year	Design	Patients	Short-term response SOT (%)	Short-term response HC (%)	Vaccine (Season), Vaccination scheme
Briggs	1980	pc	RTX, A	A/H1N1: 12/13 (92%)	15/16 (94%)	TIV (NA), single dose
				A/H3N2: 13/13 (100%)	16/16 (100%)	TIV (NA), single dose
				B: 13/13 (100%)	16/16 (100%)	TIV (NA), single dose
Huang	1983	pc	RTX, A	Overall: 14/27 (52%)	12/12 (100%)	TIV (1981/82), single dose
Grekas	1993	pu	RTX, A	A/H1N1: 18/31 (58%)	–	TIV (1989/90), single dose
				A/H3N2: 17/40 (42.5%)	–	TIV (1989/90), single dose
				B: 16/33 (48%)	–	TIV (1989/90), single dose
Kobashigawa	1993	r	HTX, A	A: 12/18 (67%)	–	TIV (1988/89), single dose
				B: 6/18 (33%)	–	TIV (1988/89), single dose
Furth	1995	pc	RTX, P	A/H1N1: 15/17 (88%)	6/7 (86%)	TIV (1993/94), single dose
				A/H3N2: 12/17 (71%)	5/7 (71%)	TIV (1993/94), single dose
				B: 13/17 (82.3%)	5/7 (71%)	TIV (1993/94), single dose
Mauch	1995	pc	RTX, LTX, RLTX, HTX, P	A: 41/50 (82%)	11/11 (90.9%)	TIV (1992/93), single dose
				B: 30/50 (60%)	7/11 (63.6%)	TIV (1992/93), single dose
Edvardsson	1996	pc	RTX, P	Overall: 45/47 (96%)	5/7 (71%)	TIV (1990/91, 1991/92), single dose
Mack	1996	pu	LTX, P	A/H1N1: 22/39 (56%)	–	TIV (1993/94), double dose (month 0,1)
				A/H3N2: 26/39 (67%)	–	TIV (1993/94), double dose (month 0,1)
				B: 22/39 (56%)	–	TIV (1993/94), double dose (month 0,1)
Blumberg a)	1996	pc	HTX, PTX, RTX, LTX, A	A/H1N1: 41/68 (60%)	26/29 (90%)	TIV (1993/94), single dose
				A/H3N2: 48/68 (71%)	16/29 (55%)	TIV (1993/94), single dose
				B: 34/68 (50%)	22/29 (76%)	TIV (1993/94), single dose
Blumberg b)	1996	pu	HTX, A	A/H1N1: N/N (53%)	–	TIV (1994/95), double dose (week 0, 3)
				A/H3N2: N/N (28%)	–	TIV (1994/95), double dose (week 0, 3)
				B: N/N (25%)	–	TIV (1994/95), double dose (week 0, 3)
Admon a)^3^	1997	pc	HTX, A	A/H1N1: 9/25 (36%)	4/8 (50%)	TIV (1989/90 and 1990/91) single (controls), double (SOT, month 0,1) or triple dose (SOT, month 0, 1, 12)
				A/H3N2: 5/25 (20%)	NN (75%)	TIV (1989/90 and 1990/91) single (controls), double (SOT, month 0,1) or triple dose (SOT, month 0, 1, 12)
				B: 2/25 (8%)	2/8 (25%)	TIV (1989/90 and 1990/91) single (controls), double (SOT, month 0,1) or triple dose (SOT, month 0, 1, 12)
Admon b)^4^	1997	pc	HTX, A	A/H1N1: 7/17 (41%)	–	TIV (1989/90 and 1990/91) single (controls), double (SOT, month 0,1) or triple dose (SOT, month 0, 1, 12)
				A/H3N2: 4/17 (24%)	–	TIV (1989/90 and 1990/91) single (controls), double (SOT, month 0,1) or triple dose (SOT, month 0, 1, 12)
				B: 2/17 (12%)		TIV (1989/90 and 1990/91) single (controls), double (SOT, month 0,1) or triple dose (SOT, month 0, 1, 12)
Admon c)^5^	1997	pc	HTX, A	A/H1N1: 12/17 (71%)		TIV (1989/90 and 1990/91) single (controls), double (SOT, month 0,1) or triple dose (SOT, month 0, 1, 12)
				A/H3N2: 11/17 (65%)		TIV (1989/90 and 1990/91) single (controls), double (SOT, month 0,1) or triple dose (SOT, month 0, 1, 12)
				B: 5/17 (35%)		TIV (1989/90 and 1990/91) single (controls), double (SOT, month 0,1) or triple dose (SOT, month 0, 1, 12)
Dengler a)	1998	pc	HTX, A	A/H1N1: 8/16 (50%)	34/35 (94.3%)	TIV (1991/92), single (controls) or double dose (SOT)
				A/H3N2: 10/16 (62.5%)	33/35 (97.1%)	TIV (1991/92), single (controls) or double dose (SOT)
				B: 6/16 (37.5%)	28/35 (80%)	TIV (1991/92), single (controls) or double dose (SOT)
Blumberg a)	1998	pu	HTX, A	A/H1N1: 4/9 (44.4%)	–	TIV (1993/1994) single dose
				A/H3N2: 5/9 (55.5%)	–	TIV (1993/1994) single dose
				B: 5/9 (55.5%)	–	TIV (1993/1994) single dose
Blumberg b)^6^	1998	ra	HTX, A	A/H1N1: 4/5 (80%)		TIV (1994/1995) double dose (week 0, 3)
				A/H3N2: 4/5 (80%)		TIV (1994/1995) double dose (week 0, 3)
				B: 5/5 (100%)		TIV (1994/1995) double dose (week 0, 3)
Fraund	1999	pc	HTX, A	A/H1N1: 79/79(100%)	106/109 (97.2%)	TIV (1996/97), single dose
				A/H3N2: 55/79 (69.3%)	104/109 (95.4%)	TIV (1996/97), single dose
				B: 25/79 (31.6%)	90/109 (82.6%)	TIV (1996/97), single dose
Burbach	1999	pc	LTX, A	A/H1N1: 57/63 (92%)	57/59 (97%)	TIV (1997/98), single dose
				A/H3N2: 57/63 (92%)	59/59 (100%)	TIV (1997/98), single dose
				59/64 (95%)	59/59 (100%)	TIV (1997/98), single dose
Kimball	2000	pc	HTX, A	Overall: 12/29 (41%)	4/5 (90%)	TIV (1998/99), single dose
Sanchez-Fructuoso	2000	pc	RTX, A	A: 23/49 (46.2%)	26/37 (69%)	TIV (1996/97), single dose
				B: 10/49 (20.5%)	17/37 (44.8%)	TIV (1996/97), single dose
Soesman	2000	pc	LTX, A	A/H1N1: 53/60 (88.3%)	44/45 (97.8%)	TIV (1997/98), double dose (week 0, 4)
				A/H3N2: 56/60 (93.3%)	45/45 (100%)	TIV (1997/98), double dose (week 0, 4)
				B: 48/60 (80%)	36/45 (81.1%)	TIV (1997/98), double dose (week 0, 4)
Kimball	2001	pc	HTX, A	Overall: 12/29 (41%)	NN (80%)	TIV (1998/99), single dose
Mazzone	2001	pc	PTX, A	A/H1N1: 13/43 (30%)	15/21 (71%)	TIV (1999/2000), single dose
				A/H3N2: 17/43 (40%)	20/21 (95%)	TIV (1999/2000), single dose
				B: 4/43 (19%)	10/21 (48%)	TIV (1999/2000), single dose
Duchini	2001	pc	LTX, A	A/H1N1: 5/25 (25%)	5/9 (56%)	TIV (1999/2000), single dose
				A/H3N2: 3/15 (15%)	8/9 (89%)	TIV (1999/2000), single dose
				B: 6/20 (30%)	5/9 (56%)	TIV (1999/2000), single dose
Wyzgal	2002	pu	RTX, A	Overall: NN/23 (86.7–100%)	–	TIV (1999/2000), single dose
Cavdar	2003	pc	RTX; A	A/H1N1: 13/17 (76%)	9/10 (90%)	TIV (1997/98), single dose
				A/H3N2: 13/17 (76%)	10/10 (100%)	TIV (1997/98), single dose
				B: 17/17 (100%)	10/10 (100%)	TIV (1997/98), single dose
Lawal	2004	pu	LTX, A	A/H1N1: 37/51 (73%)	–	TIV (2002/03), single dose
				A/H3N2: 35/51 (69%)	–	TIV (2002/03), single dose
				B: 22/51 (43%)	–	TIV (2002/03), single dose
Hayney	2004	pc	PTX, A	Overall: 29/68 (43%)	22/35 (63%)	TIV (2002/03), single dose
Ballet a)	2006	pc	RTX, A	Overall: 0/6 (0%)	7/9 (78%)	TIV (2003/04), single dose
Ballet b)	2006	pc	RTX, A	Overall: 1/9 (11%)	9/15 (60%)	TIV (2004/05), single dose
Willcocks a)^7^	2006	ra	RTX, LTX, A	A/H1N1: 7/16 (44%)	–	TIV (2004/05), single dose
				A/H3N2: 13/16 (81%)	–	TIV (2004/05), single dose
				B: 7/16 (44%)	–	TIV (2004/05), single dose
Willcocks b)^8^	2006	ra	RTX, LTX, A	A/H1N1: 11/15 (73%)	–	TIV (2004/05), single dose
				A/H3N2: 15/15 (100%)	–	TIV (2004/05), single dose
				B: 10/15 (67%)	–	TIV (2004/05), single dose
Keshtkar-Jahromi a)^9^	2008	pc	RTX, A	A/H1N1: 10/16 (62.5%)	38/40 (95%)	TIV (2006/07), single dose
				A/H3N2: 14/16 (87.5%)	37/40 (92.5%)	TIV (2006/07), single dose
				B: 14/16 (87.5%)	39/40 (97.5%)	TIV (2006/07), single dose
Keshtkar-Jahromi b)^10^	2008	pc	RTX, A	A/H1N1: 22/24 (91.7%)	38/40 (95%)	TIV (2006/07), single dose
				A/H3N2: 21/24 (87.5%)	37/40 (92.5%)	TIV (2006/07), single dose
				B: 24/24 (100%)	39/40 (97.5%)	TIV (2006/07), single dose
Scharpé^11^	2008	pc	RTX, A	A/H1N1: 153/165 (92.7%)	29/41 (70.7%)	TIV (2003/04), single or double dose
				A/H3N2: 130/165 (78.7%)	34/41 (82.9%)	TIV (2003/04), single or double dose
				B: 137/165 (82.9%)	20/41 (48.8%)	TIV (2003/04), single or double dose
Gaeta	2009	pu	LTX, A	A/H1N1: 8/16 (50%)	–	TIV (2007/08), single dose
				A/H3N2: 9/16 (56.3%)	–	TIV (2007/08), single dose
				B: 6/16 (37.5%)	–	TIV (2007/08), single dose
Evison a)^12^	2009	ra	RTX, A	Overall: 30/37 (81.1%)	–	TIV (2005/06), single dose
Evison b)^13^	2009	ra	RTX, A	Overall: 33/37 (89.2%)	–	TIV (2005/06), single dose
Birdwell	2009	pc	RTX, A	A/H1N1: 37/53 (69.8%)	88/106 (83%)	TIV (2005/2006), single dose
				A/H3N2: 37/53 (69.8%)	97/106 (91.5%)	TIV (2005/2006), single dose
				B: 46/53 (86.8%)	98/106 (92.5%)	TIV (2005/2006), single dose
Salles	2009	pu	RTX, A	A/H1N1: 34/69 (43.3%)	–	TIV (2004/2005), single dose
				A/H3N2: 25/69 (36.3%)	–	TIV (2004/2005), single dose
				B: 0/69 (0%)	–	TIV (2004/2005), single dose
Candon	2009	pc	RTX, A	A/H1N1: 6/63 (9.5%)	11/19 (57.9%)	TIV (2005/2006), single dose
				A/H3N2: 13/65 (21.5%)	11/19 (57.9%)	TIV (2005/2006), single dose
				B: 13/66 (19.7%)	5/19 (26.3%)	TIV (2005/2006), single dose
Gotoh a)	2010	pc	LTX, P	A/H1N1: 1/7 (14%)	–	TIV (2006/07), single dose
				A/H3N2: 1/7 (14%)	–	TIV (2006/07), single dose
				B: 1/7 (14%)	–	TIV (2006/07), single dose
Gotoh b)	2010	pc	LTX, P	A/H1N1: 6/13 (46%)	26/63 (42%)	TIV (2007/08), single dose
				A/H3N2: 4/13 (31%)	10/63 (16%)	TIV (2007/08), single dose
				B: 3/13 (23%)	8/63 (12%)	TIV (2007/08), single dose
Gotoh c)	2010	pc	LTX, P	A/H1N1: 6/13 (46%)	26/63 (42%)	TIV (2008/09), single dose
				A/H3N2: 4/13 (31%)	10/63 (16%)	TIV (2008/09), single dose
				B: 3/13 (23%)	8/63 (12%)	TIV (2008/09), single dose
Hojsak a)	2011	pu	LTX, P	A/H1N1: 3/17 (17.6%)	–	TIV (2004/05) single dose
				A/H3N2: 7/17 (42.2%)	–	TIV (2004/05) single dose
				B: 3/18 (16.7%)	–	TIV (2004/05) single dose
Hojsak b)	2011	pu	LTX, P	A/H1N1: 20/37 (54%)	–	TIV (2005/06) double dose
				A/H3N2: 21/37 (56.7%)	–	TIV (2005/06) double dose
				B : 18/37 (48.6%)	–	TIV (2005/06) double dose
Nailescu	2011	pc	RTX, P	A/H1N1: 29/66 (43.9%)	20/21 (95.2%)	TIV (2007/2008) single dose
				A/H3N2: 20/66 (30.3%)	12/21 (57.1%)	TIV (2007/2008) single dose
				B: 23/66 (34.8%)	12/21 (57.1%)	TIV (2007/2008) single dose

Category D: Streptococcus pneumoniae							
First author	Year	Design	Patients	Short-term response SOT (%)	Short-term response HC (%)	Long-term response SOT	Vaccine, Vaccination scheme
Fuchshuber	1996	pu	RTX, P	6/7 (86%)	–	3/7(43%)	PPV23, single dose
Dengler b)	1998	pc	HTX, A	15/15 (94%)	23/23 (100%)	–	PPV23, single dose
Gennery a)	1998	pu	HTX, P	18/33 (60%)	–	–	PPV23, single dose
Kazanciouglu	2000	pu	RTX, A	21/21 (100%)	–	–	PPV23, single dose
Blumberg^14^	2001	pc	HTX, A	32/35 (91%)^9,10^	N/35 (N%)	–	PPV23, single dose
Kumar a)^15^	2003	ra	RTX, A	16/30 (53.3%)	–	–	PPV23, single dose
Kumar b)^16^	2003	ra	RTX, A	25/30 (83.3%)	–	–	PPV23, single dose
Kumar c)^17^	2003	ra	RTX, A	22/30 (73.3%)	–	–	PCV7, single dose
Kumar d)^18^	2003	ra	RTX, A	24/30 (80%)	–	–	PCV7, single dose
Kumar a)^19^	2007	ra	RTX, A			N/N(ø 49.5%)	PPV23, single dose
Kumar b)^20^	2007	ra	–	–	–	N/N (ø 66.8%)	PCV7, single dose
Barton a)	2009	pu	HTX, P	N/31 (71.5%)	–	–	PCV7 triple dose (0, 2, 4 months), PPV23 single dose
Barton b)	2009	pu	LTX, P	N/18 (>80%)	–	–	PCV7 triple dose (0, 2, 4 months), PPV23 single dose
Barton c)	2009	pu	PTX, P	N/5 (>80%)	–	–	PCV7 triple dose (0, 2, 4 months), PPV23 single dose
Barton d)	2009	pu	RTX, P	N/27 (78%)	–	–	PCV7 triple dose (0, 2, 4 months), PPV23 single dose
Gattringer a)^21^	2011	pu	HTX, PTX, A	NN/26 (ø 43%)	–	–	PCV7 single dose
Gattringer b)^22^	2011	pu	HTX, PTX, A	NN/26 (ø 32%)	–	–	PCV7 single dose followed by PPV23 single dose

Category D: Haemophilus influenzae							
First author	Year	Design	Patients	Short-term response SOT (%)	Short-term response HC (%)	Long-term response SOT	Vaccine, Vaccination scheme
Gennery b)	1998	pu	HTX, P	28/33 (85%)	–	–	Single dose conjugate HibV
Sever	1999	pc	RTX, A	24/43 (56%)	5/7 (71%)		Single dose conjugate HibV

Category D: Neisseria meningitides							
First author	Year	Design	Patients	Short-term response SOT (%)	Short-term response HC (%)	Long-term response SOT	Vaccine, Vaccination scheme
Zlamy	2011	p	RTX, LTX, P	8/8 (100%)	–	NA	Men C vaccine conjugated

Category E: Varicella							
First author	Year	Design	Patients	Short-term response SOT (%)	Short-term response HC (%)	Long-term response SOT	Vaccine, Vaccination scheme
Zamora	1994	pu	RTX, P	11/17 (64.7%)	–	NA	Single dose
Kano	2002	pc	LTX, P	5/7 (71%)	–	–	Primary vaccination before SOT, post-transplant single dose (booster)
Chaves	2005	pu	RTX, P	4/6 (66%)	–	–	Single or double (0, 1–2 months)
Weinberg	2006	pu	LTX, ITX, P	13/15 (87%)	–	–	Single dose
Khan a)	2006	r	LTX, P	20/31 (64.5%)	–	–	Single or multiple VZVV
Shinjoh a)	2008	pu	RTX, P	9/11 (82%)			Single or multiple vaccination

Category E: Mumps, Measles, Rubella							
First author	Year	Design	Patients	Short-term response SOT (%)	Short-term response HC (%)	Long-term response SOT	Vaccine, Vaccination scheme
Rand	1993	r	LTX, P	7/18 (39%)	–	–	NA
Kano a)^23^	2002	pc	LTX, P	11/13 (85%)	–	–	Primary vaccination before SOT, post-transplant single dose (booster)
Kano b)^24^	2002	pc	LTX, P	2/2 (100%)	–	–	Primary vaccination before SOT, post-transplant single dose (booster)
Kano c)^25^	2002	pc	LTX, P	6/6 (100%)	–	–	Primary vaccination before SOT, post-transplant single dose (booster)
Khan b)	2006	r	LTX, P	19/26 (73%)	–	–	Single or multiple doses MMR
Shinjoh b)^26^	2008	pu	LTX, P	9/11 (82%)	–	–	Single or multiple doses MMR
Shinjoh c)^27^	2008	pu	LTX, P	15/15 (100%)	–	–	Single or multiple doses MMR
Shinjoh d)^28^	2008	pu	LTX, P	15/15 (100%)	–	–	Single or multiple doses MMR

Category F: Tick-borne encephalitis							
First author	Year	Design	Patients	Short-term response SOT (%)	Short-term response HC (%)	Long-term response SOT	Vaccine, Vaccination scheme
Dengler	1999	pc	HTX, A	11/31(35.5%)	29/29 (100%)	–	0, 1, 3 weeks, TBEV

Category F: Rabies							
First author	Year	Design	Patients	Short-term response SOT (%)	Short-term response HC (%)	Long-term response SOT	Vaccine, Vaccination scheme
Cramer^29^	2008	r	LTX, RTX, P	7/8 (87.5%)	–	5/5 (100%)	Active (day 0, 3, 7,14, 28) RVV combined with passive immunization
Rodriguez-Romo^30^	2011	c	RTX, A	1/1	–	1/1	Active (day 0, 3, 7, 14, 28, 38, 41, 45, 52, 66) RVV combined with passive immunization

In the column “Short-term response SOT” is the number of patients given that had a positive response >2 weeks and ≤3 months after the last vaccination while in the column “Long-term response SOT” the number of patients that kept a positive response after ≥12 months is given (exceptions are marked with number). Data presented as number of patients with vaccination response/total number of patients.

*Design*: pu - prospective uncontrolled, pc - prospective controlled, r- retrospective, ra – randomized, c-case report *Patients*: A - adult, P – paediatric, HTX- heart transplantation, RTX - renal transplantation, LTX – liver transplantation, PTX- lung transplantation, ITX – intestinal transplantation, ESRD- end stage renal disease *Response*: SOT – solid organ transplant recipient, HC - healthy control group, *Vaccine, Vaccination schedule*: T – tetanus toxoid vaccine, d – diphtheria vaccine (adult formulation with reduced antigen amount), D - diphtheria vaccine (paediatric formulation with higher antigen amount), IPV – inactivated polio vaccine, HAV – hepatitis A vaccine, rHBV - recombinant hepatitis B vaccine, TIV - trivalent inactivated influenza vaccine, PPV23- 23-valent pneumococcal vaccine, PCV7- seven-valent pneumococcal conjugate vaccine, HibV – *Haemophilus influenzae* vaccine, TBEV - tick borne encephalitis vaccine, MMR – mumps, measles, rubella vaccine, RVV – rabies virus vaccine, VZVV – varicella zoster vaccine, MMRV – mumps, measles, rubella, varicella vaccine, NA - not applicable.

1 patients receiving a chimeric monoclonal antibody against CD20.

2 patients receiving conventional immunosuppressive medication.

3 after 1st dose, controls received a different vaccination scheme compared to SOT recipients (one vs. two doses of vaccine, respectively).

4 after 2nd dose, controls received a different vaccination scheme compared to SOT recipients (one vs. two doses of vaccine, respectively).

5 after 3rd dose, controls received a different vaccination scheme compared to SOT recipients (one vs. two doses of vaccine, respectively).

6 patients were randomized to vaccine vs. no vaccine for the purpose of studying rejection.

7 patients receiving calcineurin-inhibitors.

8 patients receiving sirolimus.

9 patients receiving mycophenolate mofetil.

10 patients receiving azathioprin.

11 SOT recipients received either one or two doses of vaccine, however data for the double-dose trial are not given and stated that no difference to the single dose trial.

12 subunit vaccine.

13 virosomal vaccine.

14 for controls exact numbers were not given but stated that no difference between patients and controls.

15 response measured by enzyme-linked immunoassay (ELISA).

16 response measured by opsophagonization assay (OPA).

17 response measured by enzyme-linked immunoassay (ELISA).

18 response measured by opsophagonization assay (OPA).

19 long-term response of PPV 23 vs. PCV7 by follow up of the cohort by Kumar et al. 2003, mean continued response from patients initially vaccinated against PPV23 from varying patient numbers of ranging from 2 to 10 patients.

20 long-term response of PPV 23 vs. PCV7 by follow up of the cohort by Kumar et al. 2003, mean continued response of patients initially vaccinated against PCV7 from varying patient numbers ranging from 4 to 11 patients.

21 mean response after PCV7 only to serotypes 4, 6B, 9V, 14, 18C, 19F, 23F.

22 mean response after PCV7 followed by PPV23 to serotype 1, 5 und 7F after additional PP23 vaccination in the cohort from 23.

23 response to measles component.

24 response to mumps component.

25 response to rubella component.

26 response to mumps component.

27 response to measles component.

28 response to rubella component.

29 long-term response was accepted as 6 months after vaccination.

30 adequate response was seen but which decreased rapidly.

Numbers of SOT recipients vaccinated ranged from 1 to 165 and healthy control numbers ranged from 7 to 109. There were 47 trials on vaccination of adult SOT and 25 of paediatric SOT recipients. Most trials included renal transplant recipients (RTX, 31 articles), followed by liver transplant (LTX, 18 articles), heart transplant (HTX, 11 articles), lung transplant (PTX, 2 articles) or mixed cohorts of organ transplant recipients (9 articles). Of the vaccines studied, inactivated influenza vaccination was the most common (36 articles), followed by vaccination against *Streptococcus pneumoniae* (9 articles), hepatitis B (7 articles), tetanus (6 articles), varicella (6 articles), diphtheria (4 articles), mumps, measles, rubella (4 articles), hepatitis A (3 articles), vaccination against *Haemophilus influenzae* (2 articles), rabies vaccine (2 article), polio (1 article), *Neisseria meningitides* (1 article) and tick-borne encephalitis vaccine (1 article). Some studies investigated more than one vaccine in the same cohort. No studies were found on *Bordetella pertussis*, rotavirus, human papilloma virus vaccines or vaccines recommended for certain geographic areas ("travel vaccines" such as yellow fever, typhoid fever or Japanese encephalitis vaccines) apart from tick-borne encephalitis vaccine and rabies.

Most trials only assessed the short-term response within two weeks to three months after vaccination. Only a few also measured long-term responses at a second time point. Data on long-term response were not always extractable for all SOT recipients with a positive response at first assessment after vaccination. Most trials, despite small patient numbers, addressed vaccine safety issues. None of the trials neither on inactivated or on live vaccines, however, reported severe adverse events (SAEs) or transplant-related complication such as organ rejection after vaccination during the time period of the trial.

### Short-term Response

Category A: Vaccination against tetanus, diphtheria, polio.

#### Tetanus

We retrieved six trials investigating the response to tetanus toxoid vaccination in SOT recipients with publication dates ranging from 1995 to 2010 (Table 3, category A) [16]–[21]. Four trials were prospective uncontrolled and two prospective controlled. All trials assessed the short-term response; two trials additionally assessed the long-term response [17], [20]. Three trials included adult SOT recipients and three trials paediatric SOT recipients. All trials were performed only on RTX recipients. Patient numbers ranged from 7 to 150 in SOT recipients and 13 to 96 in the control group.

In five of six studies high response rates ranging from of 85%–100% were seen [16]–[20] with no significant difference in the response rates of SOT recipients and healthy controls in controlled trials [16], [18]. Response rates were lower in the study by Puissant-Lubrano et al., which compared conventional immunosuppression to immunosuppression with an anti-CD_20_ monoclonal antibody (61.5% vs. 30.6%, respectively) (Table 3, category A) [21].

**Figure 2:**
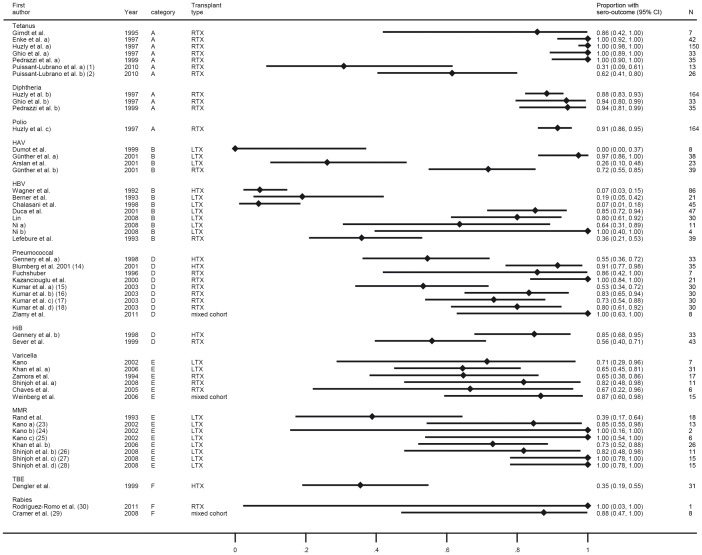
Forest plot for short-term response for vaccines from category A (tetanus, diphtheria, polio), B (hepatitis A and B), D (*Streptococcus pneumoniae*, *Haemophilus influenzae, Neisseria meningitides*), E (varicella, mumps, measles, rubella) and F (tick-borne encephalitis and rabies). All article types are included. Numbers in brackets refer to legend in Table 3.

Overall, vaccination with tetanus toxoid in the post-transplant period elicits a high rate of responders in SOT recipients with conventional immunosuppression with no significant difference to healthy controls (p = 0.96 on only two studies) while immunosuppression with anti-CD_20_ monoclonal antibody showed a decreased response rate compared to conventional immunosuppressive medication (p = 0.043 in meta-regression) (Table 3 and Figure 2, category A).

#### Diphtheria

Four trials were identified with SOT recipients vaccinated against diphtheria with publication dates ranging from 1997 to 1999 (Table 3, category A) [17]–[20]. Three trials were prospective uncontrolled while one trial was prospective controlled. Three trials were performed on paediatric SOT recipients, one trial assessed adult SOT recipients, and all were RTX recipients. Patient numbers ranged from 33 to 164 SOT recipients; the controlled trial had 106 controls.

Short-term response rates ranged from 88.5% to 95% with comparable response rates in SOT recipients and controls (88.5% vs. 96.2%, respectively) [17], [18]. All studies with diphtheria vaccination in the post-transplant phase show comparable high response rates (I-squared = 0%).

#### Polio

Only one trial with an inactivated polio vaccine (IPV) in SOT recipients was identified published in 1997 (Table 2, category A) [18]. Short-term response rate in this prospective controlled cohort of adult RTX recipients was 91.6% compared to 97.9% in controls, which shows high response for polio vaccine in the post-transplant era with no significant difference to controls (p = 0.125 based on own calculations) (see Table 3, group A).

Category B: Vaccination against viral hepatitis A and B.

#### Hepatitis A

Three trials on hepatitis A (HAV) vaccination in SOT recipients were included with publication dates ranging from 1999 to 2001, two were prospective uncontrolled and one prospective controlled (Table 3, category B) [22]–[24]. All trials were in adult recipients of either liver or kidney grafts. The trials addressed primary immunization schemes with two successive vaccine doses. Patient numbers ranged from 8 to 39 SOT recipients, the controlled trial had 27 controls. Maximal response rates after two vaccinations ranged from 0% [22], which in contrast applied a vaccination scheme with month 0 and 2 compared to all other trial with month 0 and 6, to 26% in one study [24] and 97.4% in LTX recipients and 71.8% in RTX recipients compared to 100% response in controls [23]. Overall, results for HAV vaccination show a high degree of heterogeneity (I-squared = 97.7%). While one study shows high response rates for both RTX and LTX, others did not confirm this finding (Figure 2, category B).

#### Hepatitis B

We identified seven trials on hepatitis B vaccination after SOT with publication dates ranging from 1992 to 2001 (Table 3, category B) [25]–[31]. Six trials were prospective uncontrolled while one trial was retrospective. None of the trials had a control group. Five trials were performed in LTX recipients with three in a paediatric and two in an adult LTX cohort, one trial was conducted on adult heart- and one on adult kidney transplant recipients, respectively. Patient numbers ranged from 4 to 86 SOT recipients. Most trials applied a multi dose scheme. In adult SOT recipients, the response rate was low ranging from 6.7% to 36% while in contrast, a high response rate was seen in the paediatric trials ranging from 63.6% to 100% (Figure 2, category B) [29]–[31]. Overall, response rate to HBV after transplantation in adult SOT recipients is poor while in paediatric SOT recipients a 58% (95 CI: 37%–80%) higher response rate was seen (p = 0.001 from meta-regression).

Category C: Vaccination against seasonal influenza.

In total, 36 trials on inactivated trivalent influenza vaccination after SOT were included in the review with publication dates ranging from 1980 to 2011 and vaccines against the circulating strains of the influenza season of 1981/82 to 2007/2008 [13]–[15], [32]–[64].

In nine trials a prospective uncontrolled trial design was used while 25 were prospective controlled, one trial was retrospective. Three RCT were identified which were randomized to the number of vaccine doses applied [15], to the type of immunosuppressive medication [14] and the vaccine type used [13]. Some trials applied more than one study design within one publication. Six trials investigated response in paediatric SOT recipients. 30 trials were conducted in adult SOT recipients. Patient numbers ranged from 5 to 165 for SOT recipients and from 5 to 109 for controls. Most trials were on RTX recipients (n = 19), followed by LTX (n = 11), HTX (n = 9) and PTX (n = 3) recipients.

**Figure 3:**
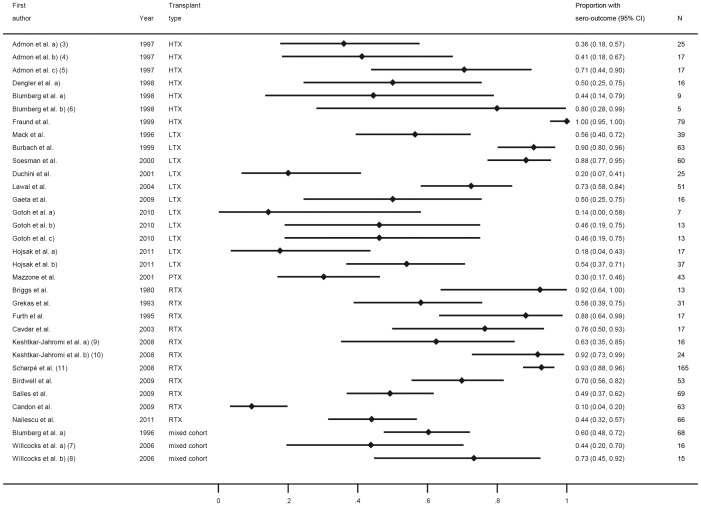
Forest plot for short-term response for vaccine category C (influenza) against influenza H1N1. All type of trials which assessed specific response to H1N1 are included. Numbers in brackets refer to legend in Table 3.

**Figure 4:**
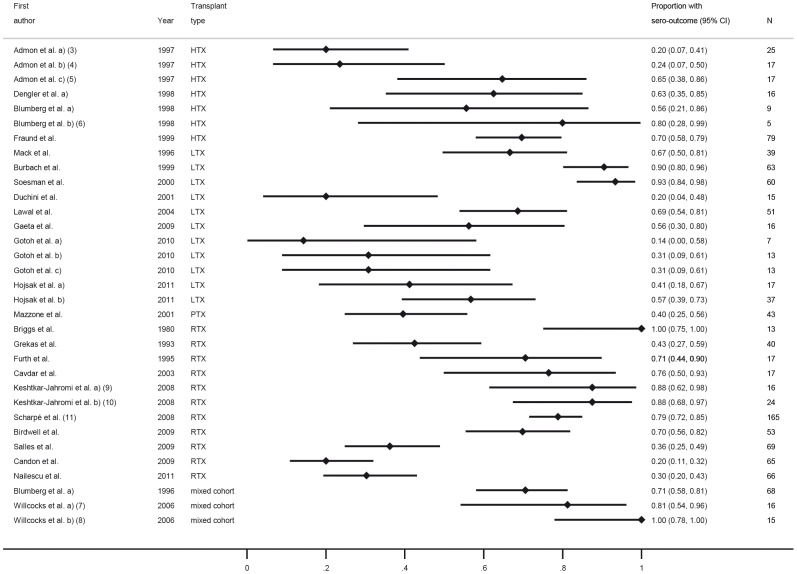
Forest plot for short-term response for vaccine category C (influenza) against influenza H3N2. All type of trials which assessed specific response to H3N2 are included. Numbers in brackets refer to legend in Table 3.

**Figure 5:**
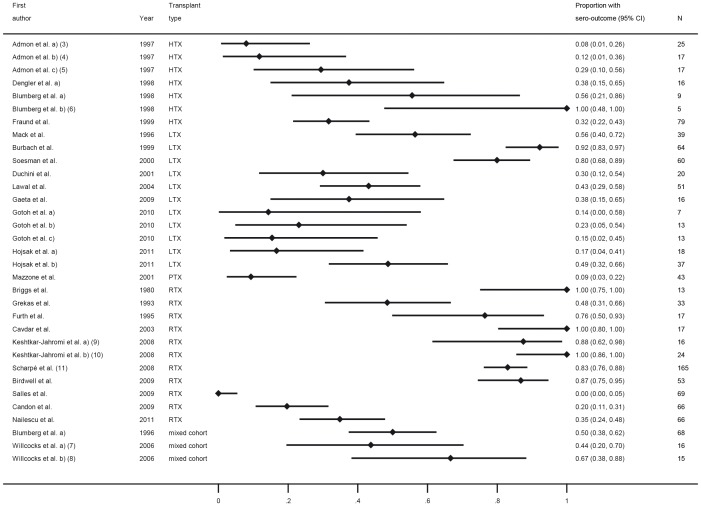
Forest plot for short-term response for vaccine category C (influenza) against influenza B. All type of trials which assessed specific response to B are included. Numbers in brackets refer to legend in Table 3.

Vaccination response was in most trials investigated as the response to each of the three components A/H1N1, A/H3N2 and B (Figure 3, 4, 5), while others only observed overall response to influenza A or B or only total overall response (the latter two type of trials are not displayed in a figure). The most common applied vaccination scheme was single dose while some trials investigated a double or even triple dose scheme of the same vaccine, however in several controlled trials the control group received a different vaccination scheme than SOT recipients (single vs. multi dose regimen, respectively). Conflicting results were found for multiple versus single dose vaccination in SOT recipients. While some trials revealed an increased response rate after multiple dose vaccination in SOT recipients, in other trials this was less clear. Furthermore, the multiple dose regimen was not applied in most control groups. None of the trials assessed long-term vaccination responses.

In the trials comparing different immunosuppressive drugs, calcineurin-inhibitors and azathioprin were associated with a slightly better response compared to sirolimus and mycophenolate mofetil (Figure 3, 4, 5) [14], [56].

**Figure 6:**
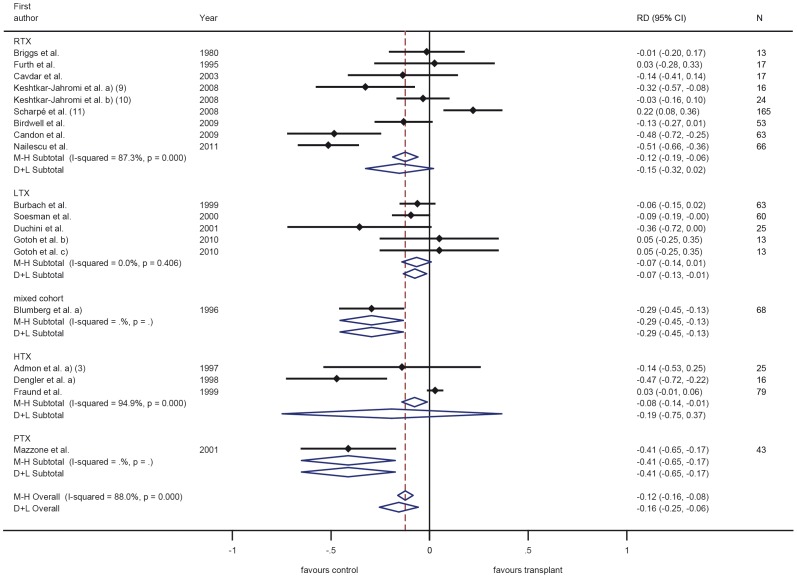
Meta-analysis for case-control studies using the Mantel-Haenszel fixed effects method (M–H) and the DerSimonian and Laird random effects (D+L) method for response to influenza H1N1. Numbers in brackets refer to legend in Table 3.

**Figure 7:**
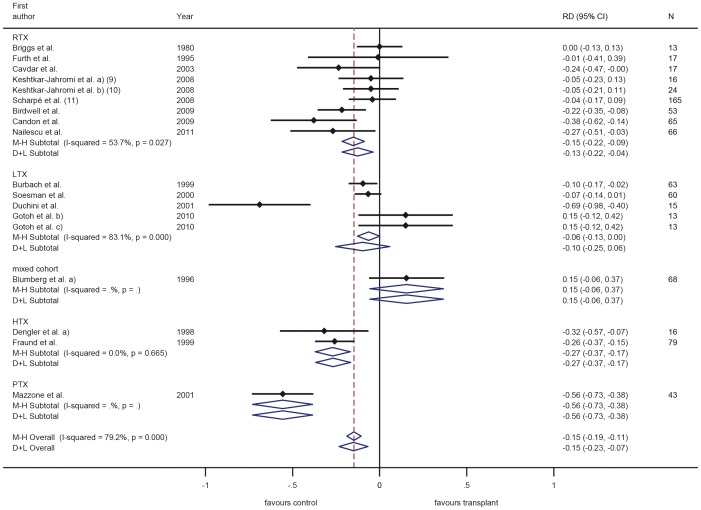
Meta-analysis for case-control studies using the Mantel-Haenszel fixed effects method (M–H) and the DerSimonian and Laird random effects (D+L) method for response to influenza H3N2. Numbers in brackets refer to legend in Table 3.

**Figure 8:**
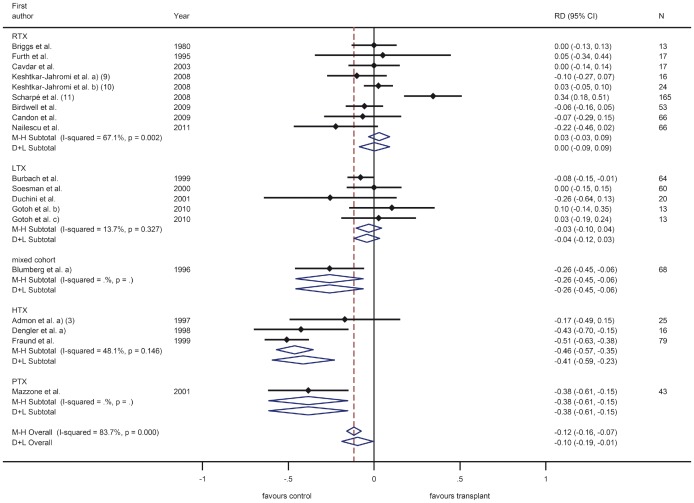
Meta-analysis for case-control studies using the Mantel-Haenszel fixed effects method (M–H) and the DerSimonian and Laird random effects (D+L) method for response to influenza B. Numbers in brackets refer to legend in Table 3.

Overall, variability of the response was very high and ranged from 0–100% in SOT recipients with corresponding high values of I-squared (all larger than 92%) (Figure 3, 4, 5). When comparing response rate of SOT recipients with controls, several studies showed a clear trend to a less pronounced response in SOT recipients with overall a 10% to 16% lower response rate in SOT recipients (Figure 6, 7, 8). The difference in response to control patients was in renal and lung transplant recipients less pronounced than in the other SOT groups. In some studies, however, the response rate in SOT recipients was even higher than in controls. Almost all trials, however, observed a measurable vaccine response at least in a subset of SOT recipients after single dose vaccination with a trend to lower response rates in SOT recipients compared to healthy controls in most of the trials.

Category D: Vaccination against bacterial pathogens.

#### Streptococcus pneumonia

Nine studies were identified which investigated pneumococcal vaccine in SOT recipients with publication dates ranging from 1998 to 2011 (Table 3, category D) [11], [12], [42], [65]–[70].

The trial design was prospective uncontrolled in five trials, and prospective controlled in two trials. Two RCTs were randomized to the type of vaccine given. Three trials were on paediatric and five on adult SOT recipients. There were trials on SOT recipients of kidney (3 trials), heart (3 trials), and three trials on mixed cohorts.

In four trials responses to a randomized or mixed vaccination scheme with both types of vaccines were investigated. Overall response ranged from 32% to 100% with comparable responses in the control group, if included [42], [68]. Response rate was assessed either as response to at least one serotype from a group of representative antigens or an overall or mean response to selected antigens. Those antigens selected as “representative” varied between trials. Response rate in all studies was above 50% with a summary estimate of 83% (95% CI: 83%–93%) with substantial heterogeneity (I-squared = 81%) (Figure 2, category D).

#### Haemophilus influenzae (Hib)

We retrieved two prospective trials on Hib vaccination in SOT recipients published in 1998 and 1999, one prospective controlled in RTX and one prospective uncontrolled in HTX recipients (Table 3, category D) [66], [71]. The vaccination responses were 56% in adult RTX recipients compared to 71% in controls [71] and 85% in paediatric HTX recipients [66] one month after vaccination with Hib conjugate vaccine. Long-term responses were not assessed. An acceptable response rate above 50% in SOT recipients in both studies was observed (Figure 2, category D).

#### Neisseria meningitides

We retrieved one study which investigated vaccination with conjugated meningitis C vaccine in a mixed cohort of paediatric SOT recipients [72]. A positive short-term response after 3 months was seen in all patients (Figure 2, category D), however the authors stated that titers waned rapidly.

Category E: Vaccination with attenuated live virus vaccines.

#### Varicella

Six trials on live-attenuated varicella vaccination after SOT were identified with publication dates ranging from 1994 to 2008 (Table 3, category E) [73]–[78]. Five were prospective uncontrolled and one was retrospective. All trials were conducted in paediatric SOT recipients with three trials on RTX recipients, [73], [74], [76] two trials on LTX [77] and one trial on a mixed cohort of recipients of liver or intestine grafts [75]. Short-term response rates ranged from 64.5% to 87%. Acceptable response rates were observed in all studies with an overall estimate of 73% (95% CI: 64%–83%) with little heterogeneity (I-squared = 0%) in SOT recipients after post-transplantation vaccination are seen (Figure 2, category E).

#### Mumps, measles and rubella

Four trials on mumps, measles and rubella vaccination in SOT recipients were identified with publication dates ranging from 1993 to 2008 [74], [77]–[79]. Two trials were retrospective and one prospective uncontrolled, none of the trials assessed long-term responses. Only two trials specified the immune response to the individual components of the vaccine. Overall, all trials had small patient numbers ranging from 6 to 26 SOT recipients with a vaccination response ranging from 39% to 100%. In the two larger retrospective studies by Khan et al. and Rand et al., which observed response rates over a time period of 15 months and 5 years, respectively, lower response rates were observed. In the trial by Shinjoh et al., high response rates in a very small patient number was seen with a lower response rate to the mumps component of the vaccine (Table 2, category E).

Overall, the observed positive response rates were above 70% in all but one study conducted in 1993 resulting in a summary estimate of 85% (95% CI: 72%–99%) with substantial heterogeneity (I-squared = 76%) (Figure 2, category E). Omitting the 1993 study reduced heterogeneity substantially (I-squared = 36%).

Category F: Vaccines indicated in certain geographical areas.

#### Tick-borne encephalitis

One trial was included that measured vaccination response after TBE vaccination, published in 1999 (Table 3, category F) [80]. In this prospective controlled trial, TBE vaccination in an endemic area in Germany was studied in 31 HTX recipients. SOT recipients responded in 35.5%, controls in 100% (Figure 2, category F).

#### Rabies

Two trials were found that assessed immune response to rabies vaccine in SOT recipients which were exceptionally included despite not meeting all inclusion criteria [81], [82] and despite the fact that in both trials active and passive vaccination was performed, due to the global relevance of rabies and the importance to know the response of SOT recipients to standard post-exposure vaccination as the only remedy after a potential risk contact (Table 3, category F). Vaccination response to the post-exposure vaccination scheme was analysed in an uncontrolled retrospective trial in 8 paediatric SOT recipients [82] and in an adult kidney transplant patient [81]. In the study by Cramer et al., the short-term response was 87.5% and in a 6 months assessment, anti-rabies antibodies were found in 100% of SOT recipients. Because of the relevance of this finding, we exceptionally included the 6-month response into our analysis. In contrast, the case report by Rodriguez-Romo et al. reported a decrease of antibodies already 28 days after vaccination after adequate levels were initially reached after the third dose of vaccine. Reduction of immunosuppression and booster doses recovered adequate antibody levels (Figure 2, category F). In both trials passive immunization may have led to an overestimation of the vaccine response. The summary response rate estimate seen in these 9 SOT recipients was 89% (95% CI: 52%–100%) (Figure 2, category F).

### Long-term Response of SOT Recipients

**Figure 9:**
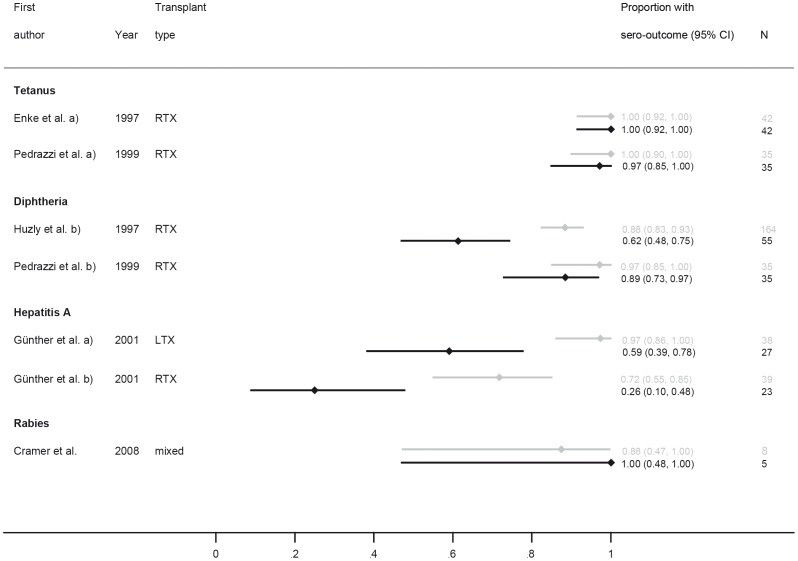
Forest plot for studies investigating both short-term response (grey bars and numbers) and long-term response (black bars and numbers).

Only few studies assessed a long-term response after ≥12 months, these were Enke et al. and Pedrazzi et al. for the tetanus trials, Huzly et al. and Pedrazzi et al. for the diphtheria, Günther et al. for hepatitis A vaccination and rabies with the caveats of passive immunization and a time period of 6 months instead of 12, Cramer et al. (Figure 9) [17], [18], [20], [23], [82]. Long-term response for tetanus showed persistence of antibodies without relevant decrease while diphtheria and polio long-time responses were decreasing (polio not included in the figure because absolute number of SOT recipients in the long-term cohort not assessable). A strong decline in antibodies after vaccination was also seen for hepatitis A vaccine with a decline 97.4% to 59.3% and 71.8% to 26.1%. Overall, based on this limited number of studies that assessed both short- and long-term response to vaccinations, the response for diphtheria had a 17% (95% CI: 7%–27%) and the one for hepatitis A 41% (95% CI: 26%–57%) decline over time in SOT recipients (Figure 9).

## Discussion

To the best of our knowledge, this is the first systematic review of the literature on vaccination response of currently licensed vaccines in SOT recipients.

The most important finding is that most clinical trials conducted and published over more than 30 years have all been small and highly heterogeneous regarding trial design, patient cohorts selected, patient inclusion criteria, dosing and vaccination schemes, follow up periods and outcomes assessed. The evidence base for vaccination recommendations for SOT recipients thus remains poor.

Our review had two main strengths: The systematic strategy and broad search terms used to identify studies in a wide range of databases and the rigorous methods used to extract and appraise the data. The main limitation of this review was the need to rely on studies that were largely observational. The potential for confounding and bias should therefore be considered when interpreting the results and the observed level of heterogeneity of the results.

The second most important finding is that the individual vaccines investigated have been studied predominately only in one group of SOT recipients, i.e. tetanus, diphtheria and polio in RTX recipients, hepatitis A exclusively in adult LTX recipients and mumps, measles and rubella in paediatric LTX recipients. There is a clear need to investigate all recommended vaccines across all SOT types. There is increasing concern about high individual and SOT-dependant variation in the response to immunosuppressive drugs in both paediatric and adult SOT recipients [83], [84].

Most trials in SOT recipients included in this review have evaluated influenza vaccines. Variability of the response was very high and ranged from 0–100% in SOT recipients with corresponding high values of I-squared (all larger than 92%). Among other reasons (such as studies conducted over more than 30 years) the varying seasonal composition of influenza vaccines may play a role and makes comparisons between trials difficult. When comparing response rates of SOT recipients with controls, several studies showed a clear trend to a less pronounced response in SOT recipients with overall a 10% to 16% lower response rate in SOT recipients. The difference in response to control patients was in renal and lung transplant recipients somewhat less pronounced than in the other SOT groups. In some studies, however, the response rate in SOT recipients was even higher than in controls, which might reflect the play of chance or the fact that in some of these studies different vaccination schemes were used in the two groups (i.e. double dose for SOT recipients and single dose for controls) or highly differing pre-transplant antibody levels (higher in SOT recipients than in controls). Almost all trials, however, observed a measurable vaccine response at least in a subset of SOT recipients after single dose vaccination with a trend to lower response rates in SOT recipients compared to healthy controls in most of the trials, which is encouraging and is in accordance with findings from other authors [85]–[87]. In contrast to other vaccinations, influenza vaccination is recommended annually to protect against the predicted seasonal strains; rapid decline of antibodies is, therefore, of marginal relevance. Given the susceptibility, morbidity and mortality associated with influenza infections in SOT recipients and the positive response found in most influenza vaccine trials, recommending annual influenza vaccination is encouraged by our review of published clinical trials. The benefit for accelerated vaccination schemes or multi dose vaccination to increase protection cannot be decided on the basis of available data.

Good evidence and encouraging results were found for vaccinating SOT recipients against tetanus, diphtheria and polio. Vaccination with tetanus toxoid in the post-transplant period elicits a high rate of responders in SOT recipients with conventional immunosuppression with no significant difference to healthy controls (p = 0.96 on only two studies) while immunosuppression with anti-CD_20_ monoclonal antibody showed a decreased response rate compared to conventional immunosuppressive medication (p = 0.043 in meta-regression). All studies with diphtheria vaccination in the post-transplant phase show comparable high response rates (I-squared = 0%). From the data reviewed serological response to tetanus appears to be longer lasting than to diphtheria vaccination. Long-term response for tetanus showed persistence of antibodies without relevant decrease while the response for diphtheria had a 17% (95% CI: 7%–27%) decline over time in SOT recipients.

Response to polio vaccination also seems to be elicitable in SOT recipients, even though only one trial has investigated this vaccine. However, as polio eradication suffers from backlashes and international travel of SOT recipients is increasing, polio vaccination in SOT recipients is important and deserves further exploration.

Vaccination trials for viral hepatitis A and B have largely been conducted in LTX recipients and results can only be extrapolated to other SOT types with great care. Results for HAV vaccination show a high degree of heterogeneity (I-squared = 97.7%), while one study shows high response rates for both RTX and LTX, others did not confirm this finding. Response rate to HBV after transplantation in adult SOT recipients is poor while in paediatric SOT recipients a 58% (95 CI: 37%–80%) higher response rate was seen (p = 0.001 from meta-regression). The results of primary vaccination after SOT are disappointing as early response rates were low in almost all trials and long-term responses have not been assessed on a large scale. In one study, however, long-term response was assessed and antibodies for HAV were declining rapidly. The response for hepatitis A had a 41% (95% CI: 26%–57%) decline over time in SOT recipients.

In the light of this finding, the importance of hepatitis A and B vaccination prior to transplantation should be markedly stressed in pre-transplant assessment and counselling. The immunogenicity of both vaccines needs to be extended beyond LTX and should be evaluated in recipients of organs other than liver.

Vaccination against bacterial pathogens is of great importance for immunosuppressed recipients. Response rate in all *Streptococcus pneumoniae* studies was above 50% with a summary estimate of 83% (95% CI: 83%–93%) with substantial heterogeneity (I-squared = 81%), for *Haemophilus influenzae vaccines (Hib)* a response rate above 50% in SOT recipients in both studies was observed as well as for *Neisseria meningitides* (100%).

Assessment of the response to pneumococcal vaccines is difficult due to the large numbers of serotypes included in the vaccines (conjugate vaccine with 7 serotypes and polysaccharide vaccine with 23 serotypes) and the unclear impact of the seroresponse measured on protection. The response rate assessed here might be overestimated as we accepted the serological response to a single antigen as positive response. However, even in healthy children and adults, vaccine-, serotype-, and population-specific differences in immune response is not readily understood [88], [89]. Most guidelines, nevertheless, recommend pneumococcal vaccines for SOT recipients. From current data it cannot be assessed if conjugate pneumococcal vaccines are superior to polysaccharide vaccines in SOT recipients.

Vaccines for protection of travel-related infections in SOT recipients have, with very few exceptions, not been studied so far. Due to increasing quality of life, SOT recipient are willing to travel and a thorough assessment of their vaccination status is therefore necessary [90], [91]. Also, it is important to note that some of these infections are highly endemic or epidemic in countries where SOTs are now also regularly performed. Rabies is an example. We could identify only a single and very small trial on rabies post-exposure prophylaxis. The summary response rate estimate seen in these nine SOT recipients was 89% (95% CI: 52%–100%). These results are encouraging for rabies vaccination in SOP recipients. From a global point of view research in this area is warranted.

Vaccination of SOT recipients with live-attenuated viral vaccines remains controversial and is clinical studies are currently limited to paediatric SOT recipients [92]–[94]. Generally, live vaccines are contraindicated in immunocompromised recipients as there is a risk of vaccine-virus replication. As all other trials identified in our review, the trials investigating live vaccines are also underpowered to assess severe adverse events (SAEs) with appropriate accuracy. For live-attenuated varicella vaccination acceptable response rates were observed in all studies with an overall estimate of 73% (95% CI: 64%–83%) with little heterogeneity (I-squared = 0%) in SOT recipients after post-transplantation vaccination are seen. For mumps, measles and rubella positive response rates were above 70% in all but one study conducted in 1993, resulting in a summary estimate of 85% (95% CI: 72%–99%) with substantial heterogeneity (I-squared = 76%). Omitting the 1993 study reduced heterogeneity substantially (I-squared = 36%). In terms of vaccination response, the trials presented here show encouraging results at least for paediatric recipients. The trials so far performed were very small, however, and do not allow to assess the risk-benefit ratio of vaccination vs. infections for e.g. measles or varicella. The underpowerment is a problem to accurately assess vaccine-related SAEs in SOT recipients in all trials conducted so far. On theoretical grounds the risk of SAE is expected to be less in inactivated compared to live-attenuated vaccines. On the basis of currently available evidence application of live vaccines should remain limited to carefully monitored trials until more data on safety are available. Instead, indirect protection of SOT recipients by vaccination of household contacts is stressed by all authors.

Vaccination aims for long-term protection after initial immunization. To which extent this can be achieved in SOT recipients is unclear. Only few studies assessed a long-term response after ≥12 months. Long-term response for tetanus showed persistence of antibodies without relevant decrease while response for diphtheria had a 17% (95% CI: 7%–27%) and the one for hepatitis A 41% (95% CI: 26%–57%) decline over time in SOT recipients. Based on this limited number of studies that assessed both short- and long-term response to vaccinations no recommendation on adaptation of general recommendations for booster intervals for SOT recipients can be given.

Limitations of our review are that, despite the use of systematic search strategies, some trials may have been missed, particularly since the search was limited to trials published in English. We did not assess the quality of the trials. All trials included in this review were, however, published in peer-reviewed journals. Only publications in which essential key data were missing were excluded. Since we did not restrict our search to a specific publication period, older trials using out-dated immunosuppressive regimens, which are no longer relevant, may have been included. We are aware that the type of immunosuppressive treatment impacts on the vaccine response. Stratification by type of immunosuppressive treatment would have, however, further fragmented the presentation of available data with the result of even smaller numbers per stratum. Equally, we did not exclude trials on the basis of the time period between SOT and vaccination despite the fact that the first 6 months after transplantation is regarded as the period during which immunosuppression is highest.

Additional potential sources of heterogeneity in positive response to vaccination were substantial loss to follow-up and often unknown and possibly varying levels of pre-transplant immunity to the infections covered in the respective vaccines. An unknown vaccination status of a SOT recipient represents, however, a situation frequently seen in clinical practice and requires a pragmatic approach.

With these caveats in mind, we believe that this review comprehensively presents the current knowledge on vaccination response in SOT recipients.

### Interpretation

Vaccine-based prevention of infectious diseases is far from satisfactorily in solid organ transplant recipients. Despite the large number of vaccination trials preformed over the past decades, knowledge on vaccination response after SOT is still limited. Even though the protection, which can be achieved in SOT recipients through vaccination, appears encouraging on the basis of available data, current vaccination guidelines and recommendations for post-SOT recipients remain poorly supported by evidence. There is an urgent need to conduct appropriately powered vaccination trials with relevant endpoints in well-defined SOT recipient cohorts, as well as to increase the awareness of clinicians for timely pre-transplant vaccination.
